# *Legionella*: Molecular Microbiology

**DOI:** 10.3201/eid1501.081248

**Published:** 2009-01

**Authors:** Thomas Marrie

**Affiliations:** University of Alberta, Edmonton, Alberta, Canada

**Keywords:** Legionella, Legionnaires’ disease, molecular microbiology, book review

The first 3 chapters of *Legionella*: Molecular Microbiology deal with clinical aspects of legionellosis. Paul Edelstein provides a fascinating glimpse into the history of Legionnaires’ disease, revealing little-known facts from the perspective of one who participated in the investigation of the 1979 outbreak at Wadsworth Veterans Administration Hospital in Los Angeles, California. Although the chapter on epidemiology focuses on Legionnaires’ disease in Europe, it provides new information from the European Working Group for *Legionella* Infections.

The remaining 9 chapters cover various aspects of the molecular biology of *Legionella*. These chapters provide up-to-date information for basic scientists, but they are also of value to clinicians. However, the chapter on genetics and immunology of host resistance to *Legionella* infections is illuminating, yet disappointing—illuminating because it shows that much has been learned but disappointing in that we still do not understand much about the biology of *Legionella* infections. It is also disappointing that the efforts of clinicians and basic scientists are not coordinated at the time of outbreaks, so material could be collected that would enable more in-depth study of the genetics and immunology of host resistance.

The book provides a glimpse into the challenges of studying an intracellular pathogen that is found in human macrophages and in amebae. For anyone who studies intracellular pathogens, the chapter on mechanisms of intracellular survival and replication of *L. pneumophila* will be instructive.

One minor distracting feature is that the introduction to most chapters tends to repeat the same material about Legionnaires’ disease. Also, a background in molecular biology would be helpful in understanding some of the more technical chapters, although it is not absolutely necessary. Overall, not only will this book be pertinent to all who study *Legionella* spp. and other intracellular microorganisms in the laboratory, but it will also be a valuable reference for infectious disease clinicians, microbiologists, and public health professionals.

